# Cancer survivorship: an integral part of Europe's research agenda

**DOI:** 10.1002/1878-0261.12428

**Published:** 2019-01-08

**Authors:** Pernilla Lagergren, Anna Schandl, Neil K. Aaronson, Hans‐Olov Adami, Francesco de Lorenzo, Louis Denis, Sara Faithfull, Lifang Liu, Franḉoise Meunier, Cornelia Ulrich, the European Academy of Cancer Sciences

**Affiliations:** ^1^ Surgical Care Science Department of Molecular medicine and Surgery Karolinska Institutet Karolinska University Hospital Stockholm Sweden; ^2^ Department of Surgery and Cancer Imperial College London UK; ^3^ Department of Quality and Patient Safety Karolinska University Hospital Stockholm Sweden; ^4^ Division of Psychosocial Research & Epidemiology The Netherlands Cancer Institute Amsterdam The Netherlands; ^5^ Department of Medical Epidemiology and Biostatistics Karolinska Institutet Stockholm Sweden; ^6^ Clinical Effectiveness Research Group Institute of Health and Society University of Oslo Norway; ^7^ European Cancer Patient Coalition Brussels Belgium; ^8^ Italian Federation of Cancer Patients Organisations Rome Italy; ^9^ Oncology Centre Antwerp Belgium; ^10^ Faculty of Health and Medical Sciences University of Surrey Guildford UK; ^11^ Fédération of European Academies of Medicine Brussels Belgium; ^12^ Huntsman Cancer Institute Salt Lake City UT USA; ^13^ Department of Population Health Sciences University of Utah Salt Lake City UT USA

**Keywords:** cancer research, comprehensive cancer centres, long‐term effects, research programmes, survivors

## Abstract

Cancer survivorship has traditionally received little prioritisation and attention. For a long time, the treatment of cancer has been the main focus of healthcare providers’ efforts. It is time to increase the amount of attention given to patients’ long‐term well‐being and their ability to return to a productive and good life. This article describes the current state of knowledge and identifies research areas in need of development to enable interventions for improved survivorship for all cancer patients in Europe. The article is summed up with 11 points in need of further focus.

**Abbreviations**
CCC: comprehensive cancer center
EORTC: European Organisation for Research and Treatment of Cancer
HRQOL: health‐related quality of life
iPAAC: innovative partnership for action against cancer
PRO: patient‐reported outcomes

## 1 Introduction

In Europe, more than 3 million new cases of cancer occur every year (Ferlay *et al*., 2013). Currently, almost one in three individuals will develop cancer during his or her lifetime (Jemal *et al*., 2011). Due to advances in early detection, improved therapies and supportive care, cancer survival rates have increased substantially over the past decades (Malvezzi *et al*., 2015). To date, about half of patients who are diagnosed with cancer will survive for 10 years or more (Allemani *et al*., 2018; Cancer Research UK, 2017, De Angelis *et al*., 2014). The proportion of people predicted to survive a diagnosis of cancer is increasing by ~ 3% per year (Guzzinati *et al*., 2018). An increasing prevalence of individuals with long‐term chronic health problems and comorbidity will require a healthcare system, which can accommodate their growing need for long‐term follow‐up, good quality of life and functioning, returning to work, living independently and a reduction of cancer recurrence.

The concept of ‘cancer survivorship’ was first articulated in 1985 by Mullan in the highly influential paper ‘Seasons of Survival’. He divided cancer survivorship into three phases: acute, extended and permanent survival (Mullan, 1985). Since then, the US institute of medicine issued its report ‘From Cancer Patient to Cancer Survivor – Lost in Transition’ (Insititute of Medicine and National Research Council, 2006), where cancer survivorship was defined to encompass the entire cancer continuum from initial diagnosis through the remainder of life. Cancer survivorship often focuses on the distinct phase of cancer care that takes place after active cancer treatment and includes physical, mental and social aspects of living with and after a cancer diagnosis.

Subsequently, substantial progress has been made in survivorship research, especially in the area of immediate, persistent and late effects of cancer treatments (Ganz *et al*., 2012). Many cancer patients suffer from early or late effects of cancer and its treatment that may cause physical and psychosocial morbidity, and premature death (Aaronson *et al*., 2014; Fang *et al*., 2010, 2012; Hewitt *et al*., 2003; Lu *et al*., 2013). However, along with the development of novel therapies, new symptoms and side effects are emerging. For example, immunotherapies have significant health and functional impact, such as heart failure and musculoskeletal dysfunction, which are not clearly defined in clinical trials (Armenian *et al*., 2017; Zamorano *et al*., 2016). Once new treatments have become a part of current clinical practice, long‐term follow‐up of patients receiving these treatments needs to be planned.

Translational cancer research aims to create a continuum from basic/preclinical to clinical and outcomes research, resulting in the adoption of new diagnostic methods and treatments. Once sufficient evidence has been accumulated to show a reasonable balance between the benefits and harms of established or novel treatments, they can be incorporated into clinical guidelines. They will then ultimately be adopted into the current clinical practice and included in national cancer plans.

In Europe, long‐term follow‐up data on the impact of treatment on physical and psychosocial functioning or health‐related quality of life (HRQOL) are still lacking for most cancer types. These long‐term data are important to provide a comprehensive understanding of the outcomes of innovative diagnostics and treatments, as well as of the quality of cancer care. Knowledge of these outcomes is important to clinicians, healthcare policymakers and to patients who are cured, have no evidence of active disease or are living with cancer as a chronic condition. In addition, many patients want to know what they can do themselves to improve their health and well‐being after diagnosis. Research on health behaviours is particularly important and timely, because the time of a cancer diagnosis can be considered a ‘teachable moment’ for successful behaviour change (Demark‐Wahnefried *et al*., 2005; McBride *et al*., 2003). Finally, many patients, when correctly categorised as ‘cured’ from their first cancer, wish to live a normal life and not to be reminded of their past, nor be marked with a stigma (Dumas *et al*., 2017).

This report describes the research areas in need of development to enable interventions to improve quality of life and survivorship for all cancer patients in Europe.

## 2 Determinants of health‐related quality of life of cancer survivors

### 2.1 Late effects of treatment

The development of more efficient but less toxic treatments is fundamental for improving outcomes for cancer survivors. Despite the high cure rate achieved with cancer surgery, some patients treated surgically suffer from long‐term side effects due to loss of organ function (Cororve Gingeret *et al*., 2014; Frey *et al*., 2014; Gartner *et al*., 2010). There is a clear trend towards the use of less invasive and burdensome surgical procedures with more organ preservation as a goal (Lefebvre *et al*., 2012; Litiere *et al*., 2012; Luketich *et al*., 2003; Mack, 2001). Nevertheless, side effects of surgery may negatively affect HRQOL in the long term as well as short term (Dorval *et al*., 1998; Johansson *et al*., 2011). When accepted as standard treatment, long‐term follow‐up of patients treated with surgery can provide valuable insights into the quality of care. This outcome information can contribute to the development and implementation of physical and psychosocial rehabilitation services, and the management of side effects and their HRQOL consequences.

Approximately 50% of all cancer patients receive radiation therapy either with curative or palliative intent (Delaney *et al*., 2005). Radiation therapy can cause a wide range of acute and late side effects, as documented in more than 59 200 publications on PubMed. Second cancers may also develop as a consequence of radiation therapy (Hauptmann *et al*., 2016; Teepen *et al*., 2018). Information on long‐term side effects and impact on HRQOL of radiation therapy is fragmented and, for some cancers, lacking altogether (Faithfull *et al*., 2015; Loos *et al*., 2013). Nevertheless, technical advances in modern radiation therapy are often aimed at providing more targeted and precise radiation fields that spare healthy tissue, and thus decrease side effects (Baumann *et al*., 2016). Long‐term follow‐up of patients who have undergone radiation therapy is required to better understand the prevalence and nature of late radiation therapy‐related side effects.

Medical oncology includes traditional chemotherapy, hormonal treatment, targeted therapy and, more recently, immunotherapy. Numerous acute side effects have been documented for all medical treatment areas reported in more than 25 900 publications on PubMed. However, information about late side effects is incomplete, in part because the follow‐up time of standard clinical trials is often limited. Most documentation relates to traditional chemotherapy and hormonal treatment (Early Breast Cancer Trialists’ Collaborative Group, 2005). Targeted drugs and immunotherapy are still in their early phase of development. Long‐term follow‐up is rare and late toxicity has still not been well documented.

It is important to emphasise that most oncological treatments are multimodal, resulting in complex and sometimes unanticipated long‐term effects that need to be monitored and, where possible, treated in a multidisciplinary manner.

Research on long‐term outcomes of patients receiving anticancer treatments would ideally be based on a complementary collaboration between clinical research and population‐based cohorts. Clinical trials which record detailed and accurate information on treatment provide the best data source to estimate the risks of treatment‐related side effects, for instance dose–response of late adverse effects (Maraldo *et al*., 2015). In the era of personalised medicine, long‐term outcome databases are increasingly in demand to understand the long‐term safety profile of newly approved drugs (Kempf *et al*., 2017).

To assess the long‐term impact of anticancer treatment from the patient's perspective, clinical researchers need to undertake long‐term follow‐up studies collecting HRQOL data from cancer survivors using questionnaires and electronic devices (van der Kaaij *et al*., 2010, 2012). One approach to collect such patient‐reported outcomes (PRO) would be to conduct long‐term follow‐up data from patients who have participated in clinical trials. However, re‐contacting patients who were treated many years earlier is logistically difficult and also raises a number of medical ethical issues (e.g. informed consent procedures). Additionally, trial participants may not necessarily represent the general survivorship population, and thus, the generalisability of the findings may be limited. An alternative approach to collecting long‐term follow‐up data from clinical trial participants is to initiate prospective observational studies based on population‐based cohorts that represent real‐world survivors. Collaborating with population scientists and their databases where patients are actively followed can greatly improve the efficiency of outcomes research (Liu *et al*., 2017, 2018; van de Poll‐Franse *et al*., 2011; Thong *et al*., 2017).

### 2.2 Health behaviours

A number of lifestyle changes can influence cancer survivors’ prognosis and well‐being. Vitamin and mineral supplement use among cancer survivors is much higher than in the general population, despite concerns over interference with cancer treatment (Holmes *et al*., 2010; Rock *et al*., 2012; Velicer and Ulrich, 2008). While the benefits of supplement use are unclear, there are strong indications that a healthy diet, reduced body weight, smoking cessation, increased physical activity, use of nonsteroidal anti‐inflammatory drugs and other factors can impact prognosis and the survivorship trajectory. For example, observational studies have estimated the risk reduction of colorectal cancer recurrence with exercise to be as high as 50% (Loos *et al*., 2013). While we await the results of clinical trials evaluating the effect of exercise training on colorectal cancer prognosis, it is clear that an assessment of health behaviours should be an integral part of research studies addressing cancer survivorship. This is also important from the patient's perspective; survivors want to know what they can contribute to improve their well‐being and chances of survival.

### 2.3 Rehabilitation

Cancer is increasingly viewed as a chronic illness, as survivors often live for many years or decades after their initial diagnosis and may continue to endure physical and psychological symptoms and functional limitations. In the context of cancer, rehabilitation has traditionally been focused on physical functioning impacted by physiological symptoms of cancer treatment. However, there is a new conceptualisation that posits that cancer rehabilitation should address all the needs of survivors, including psychological, cognitive, social, sexual and nutritional symptoms (Burg *et al*., 2015; Hunter *et al*., 2017). Successful survivorship cannot be attained without rehabilitation offered as part of comprehensive survivorship care (Liu *et al*., 2016).

Comprehensive survivorship and rehabilitation plans may be valuable in supporting cancer survivors in their return to a rewarding life. The European Commission's Joint Action on Cancer Control issued recommendations on rehabilitation and survivorship, which have been endorsed by all 17 participating EU Member States. The Joint Action recommends that psychosocial and vocational rehabilitation should take a person‐centred approach. Empirical data on cancer survivors’ risk profiles in terms of health status, comorbidity, health‐related costs and mortality are a prerequisite to organising survivorship plans, and specifically tailored activities for health promotion and health care. Thus, large‐scale and nationwide data collection is warranted [‘Innovative Partnership for Action Against Cancer (iPAAC) and European cancer information system’ (https://ecis.jrc.ec.europa.eu)].

The recently published ‘Patient Guide on Survivorship’ (https://www.esmo.org/Patients/Patient-Guides/Patient-Guide-on-Survivorship) includes a section on cancer rehabilitation and timely detection, management and treatment of tumour‐related symptoms, as well as the use of a survivorship care plan that people with cancer can use in collaboration with their healthcare team to facilitate a return to normal life. Survivorship care plans are often highly appreciated by the patients, but because there is little empirical data that supports the efficacy of survivorship care plans, additional research to assess their possible benefits is urgently needed.

Comprehensive rehabilitation is a multidisciplinary concern. A new cancer care and survivorship model that integrates all multidisciplinary areas into one rehabilitation team must be established. Policy efforts are also needed to actively engage patient advocacy groups to support equal access to quality survivorship care and rehabilitation services. Involving patients in the follow‐up and management of late effects or the rehabilitation process is a major challenge. Online programmes and e‐health may be a good alternative for educating survivors, since these tools are considered cost‐efficient and show a similar impact to more conventional interventions.

### 2.4 Physical and functional fitness

Cancer‐related fatigue is one of the most common complaints of cancer patients. Fatigue often becomes chronic, extending for years into the cancer survivorship period (Daniels *et al*., 2014; Jones *et al*., 2016; Weis *et al*., 2017). It can also be a key limitation in a patient's ability to return to a productive work life. A large number of studies (including sizeable clinical trials) have shown that exercise can play an important and effective role in reducing this debilitating condition (Cheng *et al*., 2017; Cramer *et al*., 2017; Cramp and Byron‐Daniel, 2012). The benefits of exercise have been described for a multitude of cancer types (Schmitz *et al*., 2010) and appear to be most prominent among patients with an initially low performance status (Troeschel *et al*., 2018). Research in this area continues to evaluate the most effective exercise regimens, including the impact of resistance versus endurance training, and the timing in relation to surgery and therapy.

### 2.5 Psychosocial care

Between 30% and 50% of cancer survivors may experience psychological distress significant enough to warrant professional intervention sometime during the survivorship period (Mitchell *et al*., 2011). Historically, psychosocial support has been neglected in cancer treatment (Holland and Reznik, 2005). Today however, several organisations strongly recommend the inclusion of psychosocial care across the continuum of treatment, from diagnosis, through treatment into survivorship and palliative stages of care (Associazione Italiana di Oncologia Medica and Caminiti, 2013, Jacobsen, 2009, National Breast Cancer Center and National Cancer Control Initiative, 2003; National Institutet for Clinical Excellence, 2004, Skolarus *et al*., 2014). Screening for psychological distress at the time of diagnosis is used to identify patients with needs in a timely manner (Jacobsen *et al*., 2005). Still, research about psychological distress in cancer survivors is a relatively unexplored area (Jacobsen, 2009). The impact of psychosocial care and support at diagnosis and during treatment on long‐term HRQOL is poorly understood.

To help empower patients, increasing attention is devoted to facilitating shared decision‐making (Barry and Edgman‐Levitan, 2012; Makoul and Clayman, 2006). There is a need for open and affirming patient–clinician dialogue about the illness and its treatment options (Ramfelt and Lutzen, 2005; Thorne *et al*., 2013). Studies have shown that the information exchange between clinicians and cancer patients is often suboptimal (Hawley and Jagsi, 2015; Kullberg *et al*., 2015). Little is known about the effects of patient involvement in cancer treatment decision‐making on long‐term health and psychosocial outcomes.

### 2.6 Palliative care

Palliative care aims to control physical, medical and functional symptoms, psychological and emotional problems, social, existential and spiritual needs. It is intended to be personalised; using a spectrum of treatments such as isolation, altered social relationships, socio‐economic challenges and assistance with decision‐making around end of life issues (Ferrell *et al*., 2017). Palliative care is particularly relevant for improving patients’ HRQOL, their ability to remain in the home, and avoidance of over‐treatment. Palliative care has two components; early palliative care regimens should start when cure is no longer possible but with the intention of prolonging survival. Late palliative care starts when prolongation of survival is no longer possible and is focused primarily on symptom relief. Today, increasing numbers of patients with advanced cancer can live for a relatively long time, and the disease may even be viewed as chronic (Phillips and Currow, 2010). However, palliative care has traditionally been delivered late in the course of the disease (late palliative care) to patients who are hospitalised in specialised inpatient units or as a consultative service for patients with uncontrolled symptoms (Jordhoy *et al*., 2001). To have a meaningful effect on patients’ HRQOL and on end of life care, palliative care services should be well planned and provided earlier in the course of the disease (Temel *et al*., 2010). The nature and organisation of palliative care vary widely across settings, and there is insufficient evidence regarding which programmes and interventions are optimal for relieving symptoms and for maintaining, if not improving, HRQOL.

### 2.7 How should survivorship care be organised in Europe?

Europe has no formalised indications on how survivorship care should be organised. There are many recommendations and policy efforts, but no generic practical approach has been established. The challenge is to decide how survivorship care should be organised in Europe, whether in specialised survivorship clinics as in the United States, in rehabilitation clinics as in Germany, or according to an entirely different approach. Between the European countries, there are large differences in healthcare systems and culture in relation to health care and health behaviour. These differences might make a European‐wide approach feasible. Perhaps some basic features of survivorship care should be shared across countries within Europe, but each country needs to develop approaches to cancer survivorship care that reflect its own healthcare system and cultural norms. However, all European Union citizens should have equal access to optimal survivorship care (Lawler *et al*., 2014).

In the United States, specialised multidisciplinary hospital services, sometimes referred to as Survivorship Clinics, address various aspects of survivorship care. The multidisciplinary teams at these facilities may include a wide range of providers such as physicians, nurses, dieticians, mental health professionals, social workers, physiotherapists and rehabilitation specialists. Many cancer survivors, particularly those who are relatively asymptomatic or who are considered disease‐free, will likely be seen in the primary care setting by general practitioners. Thus, general practitioners will need to be integrated as an essential part of high‐quality multidisciplinary cancer survivorship care, as mentioned in the Joint Action on Cancer Control's Work Package 8 recommendations.

In light of the ever‐growing population of cancer survivors, it is important to recognise the need to avoid overpopulating the cancer care clinics, where the emphasis is on providing primary and palliative treatment. If survivors are to be seen in primary care settings, general practitioners need to be provided with adequate training and resources to understand and manage their unique long‐term care needs. Additionally, general practitioners need to be aware of the risks associated with being a cancer survivor, such as a wide range of comorbid conditions including second cancers, and deficits in functional, emotional, social and spiritual health. Specifically, healthcare providers need training and resources to identify, screen and manage long‐term, late effects such as early‐onset cardiovascular disease, osteoporosis and other organ dysfunctions.

The American Cancer Society and the George Washington Cancer Institute, with support from the centers from disease control, have collaborated to develop a series of e‐learning cancer survivorship modules aimed directly at educating general practitioners about important cancer survivorship issues. These efforts, along with the emergence of cancer rehabilitation as a focus of comprehensive care for survivors, underscore the need for additional research and policy efforts to understand how best to care for the growing population of European cancer survivors. By expanding current research capacity with increasing pan‐European funding, these objectives can be achieved. For example, cancer registries are increasingly being used to collect data on survivors, which may help produce stronger epidemiological evidence, including information on lifestyle factors, HRQOL and socio‐economic indicators to better identify the causes of, for example, inequalities in survivorship.

As recommended in the chapter on Survivorship and Rehabilitation of the European Commission Joint Action on Cancer Control's Guide (Albreht *et al*., 2017), cancer registries should begin to collect data on other factors that impact quality of life, such as rehabilitation and capacity to return to work after the completion of treatment. Systems are needed for the surveillance of long‐term and late effects of cancer treatment (physical, psychological, cognitive, social and sexual functions).

In addition to objective measures of such symptoms, there is a need for increased inclusion of PROs in routine clinical practice and in research in order to identify the issues that are most important to survivors and their loved ones. Advances in the development, implementation and evaluation of survivorship care, including comprehensive rehabilitation services, are also needed. Specifically, rehabilitation services need to focus on helping survivors achieve healthy lifestyles to improve self‐management of symptoms and long‐term health. Healthcare providers need to be offered continued training around a wide range of survivorship care issues including screening for psychosocial distress, surveillance for recurrence and second cancers, assessment and management of long‐term late effects, communication and supportive care skills, and appropriate referral to specialised providers. Integration of e‐health solutions will improve the diffusion of interventions, particularly to those within rural areas or with limitations to their ability to utilise traditional medical care settings.

## 3 Structuring future cancer survivorship research

Cancer survivorship includes a broad range of issues and challenges. Major areas of need have been outlined in anticipation of the development of a comprehensive research agenda for European cancer survivors. Close interaction between behavioural scientists, epidemiologists, clinical investigators, and media and computer scientists is needed to fully leverage the existing resources and identify the need for others. Although the predominant factors are the diagnostic stigma, the short‐term and long‐term side effects of treatment, the course of the disease and the psychosocial environment, many other personal and societal factors are at play, such as comorbidity, stress reaction, socio‐economic status and access to the healthcare system.

### 3.1 HRQOL – a fundamental issue for cancer survivors

Patient‐reported outcomes are becoming central to the understanding of cancer survivorship, clinical outcomes and patient needs. HRQOL is multifaceted and linked to a number of factors, including the disease (and its symptoms) and the treatment (and its side effects) (Aaronson, 1988). Important dimensions of PROs include anxiety, depression, distress and self‐efficacy, among others. Increasingly, methods to assess HRQOL and other PROs are being incorporated as a standard part of clinical trials and comparative effectiveness studies (US Department of Health, Human Services Health, 2006). A number of well‐validated HRQOL measures have proven useful both in clinical research and clinical practice settings. These include, among others, the European Organisation for Research and Treatment of Cancer (EORTC) portfolio of measures (the core QLQ‐C30 questionnaire and its many condition‐specific or treatment‐specific modules) (Aaronson *et al*., 1993), and the Functional Assessment of Cancer Therapy suite of measures (Cella *et al*., 1993).

More recently, both the United States National Institute of Health (the PROMIS initiative) and the EORTC (the computer adaptive technology initiative) have developed computer‐adaptive approaches to PRO measures that facilitate rapid, efficient and accurate real‐time assessment of HRQOL and other relevant outcomes in the era of personalised medicine (Cella *et al*., 2007, 2010; Petersen *et al*., 2018). Several instruments, most notably the Impact of Cancer questionnaire (Zebrack *et al*., 2006), aim to address a range of physical and psychosocial survivorship issues. The EORTC Quality of Life Group is currently carrying out a project to adapt its HRQOL measures to the survivorship setting (van Leeuwen *et al*., 2018). Development of new computer‐adaptive approaches and applications for smartphones facilitates HRQOL assessment in a cost‐effective manner with greater precision. It also allows a much timelier and detailed monitoring of treatment side effects, mental status, need for palliation and other supportive care needs (Basch *et al*., 2017), as well as more direct advice and answers to questions via such communication.

### 3.2 Comprehensive cancer centre for improved cancer survivorship

The comprehensive cancer centre (CCC) is today the optimal organisation for therapy development and delivery of high‐quality cancer care; an organisation where cancer care is integrated with research and education. Multidisciplinary research activities should include supportive care, psychosocial oncology, rehabilitation and outcomes research. Long‐term follow‐up of patients should be a mandatory mission of the CCCs. In order to reach the critical mass for comprehensive cancer survivorship research, CCCs should interact in a network to optimise exchange and harmonisation of approaches. Systematic and standardised collection of clinical information and biobanking, including long‐term follow‐up of PROs, will facilitate translational cancer research aimed at improving cancer survivorship. This is a critical and complex issue when approaching personalised cancer medicine with possibilities to predict both antitumour effects and side effects.

### 3.3 Science policy is needed for improved cancer survivorship

The current paper has revealed three salient features of science policy needs for improved cancer survivorship. First, cancer survivorship has traditionally received little prioritisation and attention. Treatment of the cancer – with surgery, radiation therapy and medical anticancer agents – has been the main focus of our efforts as healthcare providers. It is time to increase the amount of attention given to patients’ long‐term well‐being and their ability to return to a productive and good life. Second, relatively little is known about the differences in approaches to cancer survivorship across European countries. Even less is understood about how differences in health care during the survivorship phase are influenced by age, socio‐economic status, ethnicity, urban versus rural residence and possibly other factors. Third, the area of cancer survivorship research needs greater attention and acknowledgement in the scientific literature. The emergence of journals such as the Journal of Cancer Survivorship, recent survivorship conferences, the iPAAC actions, and e‐learning and collaborative research initiatives such as that of the EORTC for the development of new survivorship measures are indicators of an increasing interest in the topic of survivorship. Compared to all other areas of cancer research, the publication of high‐quality research on cancer survivorship is sparse, although increasing. This lack is associated with the scarce resources allocated by national or international bodies for observational studies on cancer survivorship. In fact, much less funding and fewer calls are dedicated to observational clinical studies, compared to experimental studies or basic research. A dedicated framework for these issues by the EU will contribute to fulfilling the needs of a vastly growing population of cancer survivors (Albreht *et al*., 2017).

We believe that the time is ripe for a European initiative to improve cancer survivorship and HRQOL among the millions of individuals who live as cancer survivors. As already indicated, the number of survivors is expected to rise substantially over time due to demographic transition, increasing incidence of some cancers and more successful treatments that will increase cure rates and extend life expectancy. A pan‐European initiative requires two components: a comprehensive research agenda that ultimately provides the evidence base for cost‐effective, individualised management of cancer survivors; and the political will to invest in a healthcare system with resources to support cancer patients beyond the phase of primary treatment and throughout their lives.

Europe might indeed have a competitive advantage in pursuing high‐quality research on cancer survivorship because it can build on existing collaborative structures, geographic proximity, initial models (e.g. the rehabilitation system in Germany) and, in some countries, an infrastructure that facilitates complete long‐term follow‐up of individuals. Examples include the population registries in Scandinavia and cancer registries with PROs in the Netherlands and in Germany. These prerequisites are lacking in most other parts of the world. For a comprehensive cancer survivorship research programme, international collaboration is required. Funding support at the European level is necessary to set cancer survivorship research priorities as well as to develop and implement research programmes.

With this background in mind, the following focus points are proposed:

- Outcomes research with a focus on cancer survivorship should be a component of the translational cancer research continuum and integrated in all CCCs.
- Long‐term follow‐up of patients should be a target of centres involved in therapy development. This would create the linkage between detailed treatment data and issues such as HRQOL and long‐term health risks of cancer survivors and may serve as a structure for translational research to limit side effects and improve HRQOL.
- Because the cure rate among paediatric oncology patients is high, the prevalence of long‐term survivors is growing and long‐term HRQOL issues are increasingly important. The pan‐European network for survivors after childhood and adolescent cancer may be a model for adult cancer survivors.
- A CCC has an important role in researching the long‐term follow‐up of patients with a focus on outcomes research and innovations. As not all patients are treated in a CCC, research should ideally be built on a collaborative partnership involving other key stakeholders to establish a robust methodology and achieve optimal external validity. Stakeholders include patient organisations, healthcare providers, clinical research organisations and those with data based on real‐world observational cohorts. Furthermore, involvement of payers and Health Technology Assessment bodies will facilitate the implementation of research findings.
- The CCC accreditation methodologies should include analyses of research on HRQOL, long‐term (treatment‐related) adverse effects, supportive care, psychosocial oncology and rehabilitation interventions.
- The assessment of health behaviours, such as exercise, tobacco smoking, use of vitamin and mineral supplements, and diet is integral to a comprehensive evaluation of factors relevant to cancer prognosis and patient HRQOL.
- Collaboration between CCCs is required to develop and validate instruments harmonised across European countries to assess HRQOL among cancer survivors.
- Funding mechanisms for international collaborations in the area of cancer survivorship research are currently lacking. With the growing population of cancer survivors, the rapid development of new diagnostic and treatment methods and the lack of information regarding HRQOL of surviving cancer patients, identification of priorities and funding mechanisms are important science policy questions that need to be addressed. A funding mechanism that aims to investigate and improve cancer survivorship should have an emphasis on Europe, in addition to the existing national perspective, with high‐level competence to review grant applications and to fund cutting‐edge research.
- In order to better reflect real‐world conditions, survivorship, effectiveness of treatments and cancer outcomes should be investigated not only in controlled clinical trial settings, but also through clinical and population‐based observational studies with long‐term follow‐up of unselected groups of patients.
- A further technical development in the collection of PROs is warranted, including computer‐adaptive approaches and applications for smartphones, and collaboration with the computer science experts should expand in order to take advantage of the Big Data revolution (Mayer‐Schönberger and Cukier, 2013).
- Finally, societal issues, such as access to work, education, insurance, loan, mortgage and financial toxicity, faced by long‐term cancer survivors should be evaluated and prioritised in the survivorship research agenda. It is critical to communicate that for a majority of cancer patients in Europe, cancer is not a death sentence, and the social sector should play a complementary role to the health sector in improving reintegration of survivors to normal social roles and activities without discrimination. Collaborative platforms, such as the EORTC Cancer Survivorship Research Program, Your Outcome Update research protocol, EORTC Cancer Survivorship Summits and the European Cancer Patient Coalition Congress, are crucial to increase such awareness and to foster efficient research in this issue (Liu *et al*., 2016, 2018).

## Author contributions

All authors contributed to the writing of this article.

## Conflicts of interest

The authors declare no conflict of interest.
